# Safety, tolerability, pharmacokinetics, and pharmacodynamics of PF-06650833, a selective interleukin-1 receptor-associated kinase 4 (IRAK4) inhibitor, in single and multiple ascending dose randomized phase 1 studies in healthy subjects

**DOI:** 10.1186/s13075-019-2008-6

**Published:** 2019-12-05

**Authors:** Spencer I. Danto, Negin Shojaee, Ravi Shankar P. Singh, Cheryl Li, Steven A. Gilbert, Zorayr Manukyan, Iain Kilty

**Affiliations:** Immunology and Inflammation Research Unit, Pfizer Worldwide Research & Development, Cambridge, MA 02139 USA

**Keywords:** IRAK4, Pharmacokinetic, Pharmacodynamic

## Abstract

**Background:**

PF-06650833 is a potent, selective inhibitor of interleukin-1 receptor-associated kinase 4 (IRAK4). Two randomized, double-blind, sponsor-open phase 1 studies evaluated the safety, pharmacokinetics, and pharmacodynamics of single (SAD) and multiple ascending doses (MAD) of PF-06650833 immediate-release (IR) and modified-release (MR) oral formulations in healthy adult subjects.

**Methods:**

Study 1 (NCT02224651) was a 96-day, placebo-substitution, SAD study of once-daily (QD) oral PF-06650833 IR 1 to 6000 mg and MR 30 to 300 mg in fasted and fed states. Study 2 (NCT02485769) was a 14-day, placebo-controlled, MAD study of PF-06650833 IR 25 to 750 mg twice daily, IR 1000 mg four times per day, IR 330 mg three times per day, and MR 300 mg QD.

**Results:**

PF-06650833 was generally well tolerated, with no dose-limiting treatment-emergent adverse events (TEAEs) identified in either study. TEAEs were generally mild in severity, with headache, gastrointestinal disorders, and acne most commonly reported. No serious AEs or deaths were reported. A maximum tolerated dose was not established in either study. In the SAD study, food intake delayed absorption of IR 30 mg and increased total exposure by 33%. Delayed absorption was achieved with the MR formulation (*T*_max_ of 1 h versus 8 h for IR 100 mg and MR 100 mg formulations, respectively). Food had no effect on total exposure for MR 30 mg, but reduced half-life 1.8-fold and increased *C*_max_ by 62%. In the MAD study, accumulation ranged from 0.9-fold to 1.4-fold for AUC_tau_ and 0.9-fold to 1.3-fold for *C*_max_. Less than 1% of the dose was recovered unchanged in urine for all dose groups, with renal clearance ranging from 14 to 23 mL/min for IR < 750 mg and MR 300 mg. There was a sustained decrease in serum high-sensitivity C-reactive protein for IR ≥ 250 mg and MR 300 mg. Based on the cholesterol/hydroxycholesterol ratio, no apparent CYP3A induction or inhibition was observed.

**Conclusions:**

PF-06650833, the first IRAK4 inhibitor to enter clinical development, has a favorable safety and pharmacokinetic profile and has shown evidence of pharmacological effect. The data support continued evaluation in human clinical trials for the treatment of rheumatic and autoimmune diseases.

**Trial registration:**

Clinicaltrials.gov, NCT02224651, registered 25 August 2014; NCT02485769, registered 30 June 2015

## Background

Autoimmune diseases such as rheumatoid arthritis (RA) and systemic lupus erythematosus (SLE) represent a continuing burden worldwide [1, 2]. In the Global Burden of Disease 2010 study, RA was ranked as the 42nd highest contributor to global disability, and other musculoskeletal disorders, including SLE, were ranked among the top 10 contributors [1, 2]. While the current estimated global prevalence is 0.24% for RA [1], and up to 0.24% for SLE [3, 4], it is expected that the number of people affected by these diseases will rise with aging populations and declining mortality rates [1, 2].

The primary goal of treating-to-target in autoimmune diseases is to achieve clinical remission or sustained low disease activity when remission cannot be achieved (such as in patients with long disease duration) [5, 6]. However, remission is often an unrealized target in clinical practice for patients with RA and SLE, with remission rates as low as 6.5–8.6% and 1.7% reported, respectively [3, 7, 8]. In addition, despite treatment, patients with RA and SLE often experience residual pain, fatigue, and impaired physical functioning [9]. Clearly, there remains an unmet medical need for therapies that advance the goal of achieving remission in all patients.

Interleukin (IL)-1 receptor-associated kinase 4 (IRAK4) is an essential signal transducer downstream of the IL-1 family receptors (IL-1R, IL-18R, and IL-33R) and the Toll-like receptors (TLRs) [10, 11]. TLRs detect bacterial and viral pathogens and may be activated by immune complexes, such as anti-citrullinated protein/peptide autoantibodies in RA [12, 13] and nucleic acid immune complexes in SLE [14, 15].

PF-06650833 is a selective, highly potent, small molecule, reversible inhibitor of IRAK4 [16]. Prior in vitro and in vivo studies have reported the inhibition of TLR-induced inflammation by small molecular inhibitors of IRAK4, including PF-06650833 [16–18]. Inhibition of IRAK4 blocks the production of inflammatory cytokines in human monocytes, including type I interferons, IL-1, IL-6, IL-12, and tumor necrosis factor, which are key drivers of autoimmune and inflammatory diseases in response to immune complex activation [17, 19, 20]. Therefore, IRAK4 is an attractive therapeutic target for diseases associated with dysregulated inflammation, such as RA, SLE, spondyloarthritis, and psoriatic arthritis.

Here, we report results from two phase 1 clinical studies in healthy subjects that evaluated the safety, tolerability, pharmacokinetics (PK), and pharmacodynamics (PD) of single (SAD) and multiple ascending doses (MAD) of PF-06650833 administered orally as immediate-release (IR) and modified-release (MR) formulations. These studies also provide preliminary evaluations of the effects of food on exposure. To our knowledge, PF-06650833 is the first IRAK4 inhibitor to enter clinical development and report safety, tolerability, PK, and PD results, which support continued exploration of IRAK4 inhibition in the treatment of rheumatic and other autoimmune diseases.

## Methods

### Study objectives

The primary objective of study 1 was to determine the safety and tolerability of SAD of orally administered IR and MR formulations of PF-06650833 in healthy adult subjects. Secondary objectives were to evaluate the plasma PK profiles of these formulations and to provide a preliminary assessment of the effect of food on the plasma PK profile of PF-06650833 (after fasting or a high-fat meal).

The primary objective of study 2 was to determine the safety and tolerability of MAD of orally administered IR and MR formulations of PF-06650833 after a standard meal in healthy adult subjects. The secondary objective was to evaluate the plasma and urine PK profiles of PF-06650833 after repeat dosing. Exploratory objectives were to assess the effects of PF-06650833 on exploratory biomarkers of PD activity, including high-sensitivity C-reactive protein (hsCRP), and to evaluate cholesterol/hydroxycholesterol ratios as an endogenous marker for cytochrome P450 3A (CYP3A) induction or inhibition [21].

### Study design and treatment

#### Study 1: SAD

**Fig. 1:**
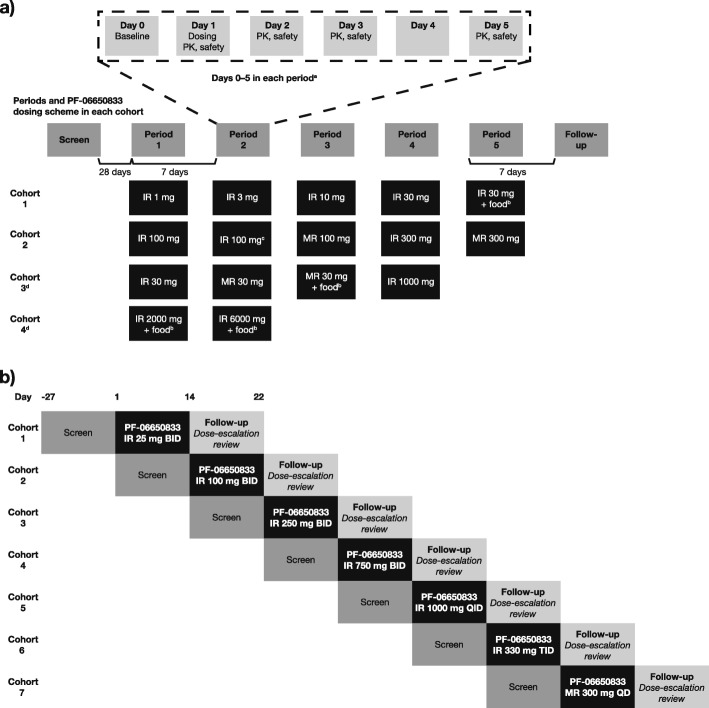
Design and PF-06650833 final dosing scheme in **a** study 1 (SAD) and **b** study 2 (MAD). ^a^PK and PD sampling time was up to 96 h for cohorts 1 and 2. Subjects in cohorts 3 and 4 were followed up to day 21 of the final period to better characterize the terminal phase, given the potentially long elimination half-life based on emerging data. ^b^Dose administered after consumption of a high-fat breakfast meal. ^c^Alternate IR formulation. ^d^Cohort 3 consisted of only four periods, and cohort 4 consisted of only two periods that were separated by 14 days, in order to maintain the overall predicted exposure in an individual subject to ≤ 28 days. In study 1, within each period, 8 subjects were randomized to receive PF-06650833 and 2 subjects were randomized to receive placebo. All subjects within a cohort received one or more doses of PF-06650833 and/or placebo. Doses were escalated sequentially within each period, based on evaluation of ≥ 48 h of safety and tolerability for all subjects and ≥ 8 h of PK data for at least 6 subjects receiving PF-06650833 and 1 subject receiving placebo. All doses were administered orally under fasting conditions (overnight fast of ≥ 10 h) unless otherwise indicated. In study 2, within each cohort, eight subjects were planned to receive PF-06650833 and 2 subjects were planned to receive placebo. All doses were administered orally under standard (not high-fat) meal, fed conditions. QD doses were 24 h apart, BID doses were 12 h apart, TID doses were 8 h apart, and QID doses were 6 h apart. When dosing in the fed condition, the morning and evening doses were administered within 5 min of completing the standard meal. *BID* twice daily; *IR* immediate-release: *MAD* multiple ascending doses; *MR* modified-release; *PK* pharmacokinetics; *QD* once daily; *QID* four times per day; *SAD* single ascending doses; *TID* three times per day

Study 1 (B7921001; NCT02224651) was a 96-day, phase 1, within-cohort, randomized, double-blind, sponsor-open, placebo-substitution, five-period crossover, SAD study in healthy adult subjects (Fig. 1a). Subjects were sequentially enrolled into four cohorts of ten subjects each. Within each cohort, the subjects were randomized into a maximum of five periods. Within each period, eight subjects and two subjects were randomized to receive PF-06650833 and placebo, respectively. In this placebo-substitution design, all subjects within a cohort received one or more doses of PF-06650833 and/or placebo. Single oral doses of PF-06650833 IR formulations from 1 to 6000 mg, and MR formulations from 30 to 300 mg, were administered in fasted (overnight fast of ≥ 10 h) and/or fed (high-fat breakfast meal) states. Doses were escalated sequentially by period within each cohort (see Fig. 1a for final dosing scheme), based upon the evaluation of ≥ 48 h of safety and tolerability for all subjects and ≥ 8 h of PK data for at least six subjects receiving PF-06650833 and one subject receiving placebo. Dose escalation was to cease when either the limits of safety and/or tolerability were reached, the projected exposure at the subsequent dose exceeded the toxicokinetic limit (TK) established based on the no observable adverse effect level (NOAEL) in relevant animal studies, or a plateau in exposure was reached.

The effects of food on the plasma PK profile of the IR and MR formulations were explored in cohorts 1 and 3, respectively, by administering 30 mg doses of PF-06650833 in a fasted state and after a high-fat meal. A 1000-mg dose of the IR formulation was also tested in cohort 3 in an attempt to identify a maximum tolerated dose (MTD). Since TK limits were not reached with the IR formulation at doses up to 1000 mg, the IR doses were escalated to 2000 and 6000 mg with a high-fat meal in cohort 4 (Fig. 1a).

#### Study 2: MAD

Study 2 (B7921002; NCT02485769) was a 14-day, phase 1, randomized, double-blind, sponsor-open, placebo-controlled, sequential group, MAD study in healthy adult subjects (Fig. 1b).

Subjects were enrolled sequentially into seven cohorts. Within each cohort, eight subjects and two subjects were randomized to receive PF-06650833 and placebo, respectively. Doses for all cohorts were administered after a standard (not high-fat) meal. Multiple dosing regimens (once daily [QD] 24 h apart, twice daily [BID] 12 h apart, three times per day [TID] 8 h apart, and four times per day [QID] 6 h apart) were used to provide required total daily doses. In the final dosing scheme, multiple oral doses of PF-06650833 IR suspension formulations at 25, 100, 250, and 750 mg BID and 1000 mg QID were administered in cohorts 1–5. PF-06650833 IR 330 mg TID (cohort 6) and an MR tablet at a dose of 300 mg QD (cohort 7) were also evaluated (see Fig. 1b for final dosing scheme).

Dose escalation (cohorts 1–5) or dose selection (cohorts 6–8) was based upon the evaluation of ≥ 7 days of safety and tolerability for all subjects and ≥ 12 h of PK data for at least six subjects receiving PF-06650833. To establish an MTD, dose escalation was to cease when the limit of tolerability was achieved.

### Randomization

In both studies, subjects were assigned to dose groups according to a pre-defined randomization schedule (see Additional file 1: Supplemental Methods). In both studies, study sponsor treatment administrators were blinded to the treatment allocation, and additional study sponsor personnel (for example, analytical staff, medical monitors, clinicians, statisticians, and pharmacokineticists) were unblinded to subject treatment allocation to permit real-time interpretation of the safety and PK data and to provide information necessary for dose escalation decisions.

### Subjects

In both studies, healthy male and female (of non-childbearing potential) subjects aged 18–55 years were eligible to participate. Subjects were to abstain from all medications (prescription, non-prescription, and/or dietary supplements) within 7 days or five half-lives (whichever was longer) prior to the first dose of PF-06650833 or placebo, except medications for the treatment of adverse events (AEs). Where possible, treatments for AEs were to avoid the use of moderate/strong inhibitors or inducers of CYP3A4. Herbal supplements and hormone replacement therapies must have been discontinued 28 days prior to the first dose of PF-06650833 or placebo. As an exception, acetaminophen/paracetamol was permitted at doses of ≤ 1 g/day. Exclusion criteria also included any clinically significant comorbid disease, screening supine blood pressure ≤ 100 or ≥ 140 mmHg (systolic) or ≤ 50 or ≥ 90 mmHg (diastolic), screening pulse or heart rate > 100 beats/min, or active or latent infection (including tuberculosis, HIV, and hepatitis viruses).

### Assessments

For both studies, samples of blood (and urine in study 2) were taken for evaluation of PK/PD parameters (see Additional file 1: Supplemental Methods). PK samples were obtained at the nominal time relative to dosing (± 10%). Plasma and urine samples were analyzed using validated, sensitive, and specific liquid chromatography tandem mass spectrometric methods (at Pfizer Inc., Groton, CT, USA, for study 1 and at Worldwide Pharmacokinetics, Dynamics and Metabolism, Cambridge, MA, USA, for study 2). The lower limit of quantification for PF-06650833 in plasma was 0.0500 ng/mL. The lower limit of quantification for PF-06650833 in urine was 1.00 ng/mL. Serum samples were also analyzed for hsCRP, and plasma samples were analyzed for 4β-hydroxycholesterol and cholesterol.

Safety assessments included incidence and severity of treatment-emergent AEs (TEAEs) and discontinuation due to TEAEs, incidence of treatment-emergent clinical laboratory abnormalities, and proportion of subjects meeting with pre-defined criteria for potential clinical concern in vital signs and electrocardiogram (ECG) parameters.

### PK and safety analyses

Sample sizes were based on clinical considerations (estimated number required to provide safety, tolerability, and pharmacological information and to minimize exposure to healthy subjects at each dose level) rather than statistical considerations. For study 1, the required total sample size was approximately 40 subjects (10 per cohort); for study 2, the required total sample size was approximately 80 subjects (10 per cohort). In study 1, a sample size of 6 subjects was sufficient to provide > 90% power to detect a food effect-related 2-fold increase of maximum observed concentration (*C*_max_) or area under the concentration-time profile curve (AUC) and 80% power to detect a 1.6-fold increase in both PK parameters for all doses except 1000 mg, assuming that the predicted within-subject PK variability would not change with food intake.

The safety analysis set was defined as all subjects who received at least one dose of study treatment, and data were summarized descriptively. The PK concentration analysis set was defined as all enrolled, treated subjects who had at least one concentration in at least one treatment period. The PK parameter analysis set was defined as all enrolled, treated subjects who had at least one of the PK parameters of interest in at least one treatment period. The PD analysis set was defined as all enrolled subjects who received at least one dose of PF-06650833 and had at least one PD parameter. PK/PD data were summarized descriptively.

PK parameters were calculated using non-compartmental analysis of concentration-time data. For study 1 only, data from cohort 1 (period 4 [IR 30 mg] versus period 5 [IR 30 mg + food]) and cohort 3 (period 2 [MR 30 mg] versus period 3 [MR 30 mg + food]) were used to estimate the effect of food intake. *C*_max_ and AUC were calculated for each subject and treatment, with and without food, after which a within-subject difference could be found between fed (high-fat meal) and fasted doses. Analysis of covariance was used to model these within-subject differences and to calculate 95% confidence intervals (CIs) for the differences for each dose (IR 30 mg and MR 30 mg). For study 2 only, the amounts of PF-06650833 in urine and renal clearance were listed and summarized descriptively. As part of the routine safety laboratory monitoring, the presence of any atypical crystals in urine specimens was semi-quantitatively assessed microscopically by typical histopathologic methods and scored as numbers of crystals per high power field (hpf); higher abundance (moderate or many) was defined as ≥ 15/hpf. No formal interim analyses were conducted.

### Ethical considerations and consent

These studies were conducted in accordance with the International Ethical Guidelines for Biomedical Research Involving Human Subjects, the Declaration of Helsinki, and the Good Clinical Practice Guidelines, along with applicable local regulatory requirements and laws. The study protocols were approved by the Institutional Review Boards and/or Independent Ethics Committee at each study center. All subjects provided written, informed consent.

## Results

### Subjects

In study 1, a total of 40 subjects were randomized and received at least 1 dose of PF-06650833, and 31 subjects received placebo. In terms of gender and ethnicity, all subjects were male, with 52.5% black, 17.5% white, 5.0% Asian, and 25.0% of another race. Mean (standard deviation (SD)) age was 38.5 (8.8) years, and all subjects were similar in regard to weight (mean [SD] 83.7 [11.8] kg), body mass index (BMI; 26.4 [3.3] kg/m^2^), and height (178.1 [6.5] cm). All treated subjects (*N* = 40) were evaluated for safety and PK.

In study 2, a total of 71 subjects were randomized; 56 subjects received PF-06650833 (IR or MR) at doses ranging from 25 mg BID to 1000 mg QID, and 15 subjects received placebo. In terms of gender and ethnicity, the majority of the subjects were male (97.2%), with 45.1% black, 22.5% white, 1.4% Asian, and 31.0% of another race. Mean (SD) age was 35.1 (8.2) years, and all subjects were similar in regard to weight (mean [SD] 82.0 [9.9] kg), BMI (26.2 [2.7] kg/m^2^), and height (177.0 [7.0] cm). All treated subjects (*N* = 71) were evaluated for safety and PK.

### Safety

#### Discontinuations

In study 1, one subject was discontinued and withdrawn per investigator request on the first day of administration due to pre-existing benign ethnic neutropenia after receiving a single dose of PF-06650833 IR 10 mg, since this would have complicated the interpretation of safety data.

In study 2, four subjects were discontinued: one each in the placebo, PF-06650833 IR 750 mg BID, IR 1000 mg QID, and MR 300 mg QD dose groups. The subjects in the placebo and MR 300 mg QD dose groups were no longer willing to participate in the study, and two subjects were discontinued due to treatment-related TEAEs as described below.

One subject in the IR 750 mg BID dose group of study 2 was discontinued on day 8 due to decreased appetite, after a prolonged period (beginning after the first dose of study drug on day 1) of pronounced gastrointestinal complaints and symptoms. The subject experienced mild nausea soon after dosing on day 1 that was exacerbated by food intake, and the subject had frequent episodes of vomiting after meals. The nausea became moderate after several days of dosing and was accompanied by moderate lack of appetite until the subject was discontinued. The TEAE was considered treatment-related, but other etiologies, such as viral gastroenteritis, could not be excluded. No other subject in the same or next higher (1000 mg PF-06650833) dose group demonstrated similar nausea symptoms.

One subject in the IR 1000 mg QID dose group of study 2 was discontinued on day 7 due to neutropenia. The subject’s absolute neutrophil count (ANC) declined from 2000/mm^3^ at screening and 1600/mm^3^ at the time of randomization to 900/mm^3^ on day 4, 1400/mm^3^ on day 5, and 1300/mm^3^ on day 7, which met the individual subject discontinuation criterion of ANC < 1500/mm^3^ on two consecutive scheduled measurements. The TEAE was considered treatment-related due to a temporal relationship with initial PF-06650833 dosing; however, further review revealed a history prior to study entry of low ANC in the subject. Although a causal relationship to PF-06650833 cannot be excluded, it is possible that the observed fluctuation in ANC was due to the previously undiagnosed cyclic (benign ethnic) neutropenia.

All nine remaining subjects in cohort 5 (IR 1000 mg QID dose group) were electively discontinued after the second dose on day 9 due to the observation of higher abundance (> 15/hpf) atypical crystals in the urine of four of these subjects on day 7. All subjects were asymptomatic and demonstrated no clinical or laboratory evidence of renal injury. These subjects were followed for safety, underwent early termination visit assessments on day 10, and were discharged from the study after follow-up on day 22.

#### Adverse events

In study 1, more all-causality TEAEs were reported with higher dose levels of IR PF-06650833, but no clear dose relationship was observed at doses ≥ 1000 mg (Table 1). The highest number of TEAEs occurred in subjects who received the IR 100 mg formulation (11 TEAEs in 3 subjects) and in subjects who received placebo (6 TEAEs in 4 subjects). Four TEAEs in 2 subjects were reported with the 300 mg MR formulation, while no TEAEs were reported with the 300 mg IR formulation. A total of 10 treatment-related TEAEs were reported in 4 subjects receiving IR PF-06650833, including 1 reporting acne (IR 30 mg; fed state), 2 reporting headache (IR 2000 mg and 6000 mg; both fed state), and 1 whose reported TEAEs, numbering 7 overall, included multiple gastrointestinal disorders (IR 100 mg; fasted). No treatment-related TEAEs were reported in subjects receiving PF-06650833 MR formulations or placebo.

**Table 1:** Study 1 (SAD) TEAEs. All causalities (treatment-related)

	Placebo^a^	PF-06650833 dose group														
		IR 1 mg	IR 3 mg	IR 10 mg	IR 30 mg	IR 30 mg (fed)	IR 100 mg	IR 100 mg^b^	IR 300 mg	IR 1000 mg	IR 2000 mg (fed)	IR 6000 mg (fed)	MR 30 mg	MR 30 mg (fed)	MR 100 mg	MR 300 mg
Subjects evaluable for AEs	31	8	8	8	15	8	8	8	8	8	8	8	8	8	8	8
Subjects with AEs	4 (0)	1 (0)	0	0	0	1 (1)	1 (0)	2 (1)	0	2 (0)	3 (1)	2 (1)	0	0	1 (0)	2 (0)
Number of AEs	6 (0)	1 (0)	0	0	0	1 (1)	1 (0)	10 (7)	0	3 (0)	4 (1)	2 (1)	0	0	1 (0)	4 (0)
Number of subjects with AEs by system organ class and preferred term																
Ear and labyrinth disorders	1 (0)	0	0	0	0	0	0	0	0	0	0	0	0	0	0	0
Vertigo	1 (0)	0	0	0	0	0	0	0	0	0	0	0	0	0	0	0
Gastrointestinal disorders	1 (0)	0	0	0	0	0	0	1 (1)	0	0	1 (0)	0	0	0	0	1 (0)
Abdominal discomfort	0	0	0	0	0	0	0	0	0	0	0	0	0	0	0	1 (0)
Abdominal distension	0	0	0	0	0	0	0	0	0	0	1 (0)	0	0	0	0	0
Abdominal pain	0	0	0	0	0	0	0	1 (1)	0	0	0	0	0	0	0	0
Diarrhea	0	0	0	0	0	0	0	1 (1)	0	0	0	0	0	0	0	1 (0)
Dry mouth	0	0	0	0	0	0	0	1 (1)	0	0	0	0	0	0	0	0
Enterocolitis	1 (0)	0	0	0	0	0	0	0	0	0	0	0	0	0	0	0
Flatulence	0	0	0	0	0	0	0	1 (1)	0	0	0	0	0	0	0	0
General disorders and administration site conditions	1 (0)	0	0	0	0	0	0	1 (1)	0	0	0	0	0	0	0	0
Fatigue	0	0	0	0	0	0	0	1 (1)	0	0	0	0	0	0	0	0
Pain	1 (0)	0	0	0	0	0	0	0	0	0	0	0	0	0	0	0
Infections and infestations	0	1 (0)	0	0	0	0	0	0	0	0	1 (0)	0	0	0	0	0
Conjunctivitis	0	1 (0)	0	0	0	0	0	0	0	0	0	0	0	0	0	0
Folliculitis	0	0	0	0	0	0	0	0	0	0	1 (0)	0	0	0	0	0
Upper respiratory tract infection	0	0	0	0	0	0	0	0	0	0	1 (0)	0	0	0	0	0
Injury, poisoning, and procedural complications	0	0	0	0	0	0	0	0	0	1 (0)	0	0	0	0	0	0
Fall	0	0	0	0	0	0	0	0	0	1 (0)	0	0	0	0	0	0
Metabolism and nutrition disorders	0	0	0	0	0	0	0	1 (1)	0	0	0	0	0	0	0	0
Decreased appetite	0	0	0	0	0	0	0	1 (1)	0	0	0	0	0	0	0	0
Musculoskeletal and connective tissue disorders	1 (0)	0	0	0	0	0	0	0	0	1 (0)	0	0	0	0	0	1 (0)
Arthralgia	0	0	0	0	0	0	0	0	0	1 (0)	0	0	0	0	0	0
Musculoskeletal stiffness	0	0	0	0	0	0	0	0	0	0	0	0	0	0	0	1 (0)
Neck pain	1 (0)	0	0	0	0	0	0	0	0	0	0	0	0	0	0	0
Nervous system disorders	1 (0)	0	0	0	0	0	0	1 (1)	0	1 (0)	1 (1)	2 (1)	0	0	0	0
Dizziness	0	0	0	0	0	0	0	1 (1)	0	0	0	1 (0)	0	0	0	0
Headache	1 (0)	0	0	0	0	0	0	0	0	1 (0)	1 (1)	1 (1)	0	0	0	0
Psychiatric disorders	0	0	0	0	0	0	0	0	0	0	0	0	0	0	1 (0)	0
Anxiety	0	0	0	0	0	0	0	0	0	0	0	0	0	0	1 (0)	0
Respiratory, thoracic, and mediastinal disorders	0	0	0	0	0	0	0	1 (0)	0	0	0	0	0	0	0	0
Hypopnea	0	0	0	0	0	0	0	1 (0)	0	0	0	0	0	0	0	0
Skin and subcutaneous tissue disorders	1 (0)	0	0	0	0	1 (1)	1 (0)	1 (0)	0	0	0	0	0	0	0	1 (0)
Acne	0	0	0	0	0	1 (1)	0	0	0	0	0	0	0	0	0	0
Dermatitis contact	1 (0)	0	0	0	0	0	1 (0)	0	0	0	0	0	0	0	0	0
Erythema	0	0	0	0	0	0	0	1 (0)	0	0	0	0	0	0	0	0
Scab	0	0	0	0	0	0	0	0	0	0	0	0	0	0	0	1 (0)
Skin irritation	0	0	0	0	0	0	0	1 (0)	0	0	0	0	0	0	0	0

All doses were administered orally under fasting conditions (overnight fast of ≥ 10 h) unless otherwise indicated. Fed doses were administered after consumption of a high-fat breakfast. Subjects were counted only once per treatment in each row. The table includes all data collected since the first dose of study drug

*AE* adverse event, *IR* immediate-release, *MR* modified-release, *SAD* single ascending doses, *TEAE* treatment-emergent AE

^a^Represents placebo groups (IR placebo, IR fed placebo, MR placebo, and MR fed placebo) in all cohorts

^b^Alternate IR formulation

In study 2, a total of 62 all-causality TEAEs were reported in 35 subjects, with PF-06650833 IR 750 mg BID having the highest number of TEAEs (*n* = 17), followed by IR 1000 mg QID (*n* = 11) and placebo (*n* = 9) (Table 2). No clear dose or regimen relationship was observed with respect to the number of TEAEs. A total of 36 treatment-related TEAEs were reported in 20 subjects, with headache, nausea, upper abdominal pain, and acne being the most common.

**Table 2:** Study 2 (MAD) TEAEs. All causalities (treatment-related)

	Placebo^a^	PF-06650833 dose group						
		IR 25 mg BID	IR 100 mg BID	IR 250 mg BID	IR 750 mg BID	IR 1000 mg QID	IR 330 mg TID	MR 300 mg QD
Subjects evaluable for AEs	15	8	8	8	8	8	8	8
Subjects with AEs	6 (2)	3 (1)	4 (2)	3 (2)	5 (5)	7 (4)	3 (2)	4 (2)
Number of AEs	9 (3)	3 (1)	5 (2)	9 (7)	17 (13)	11 (6)	4 (2)	4 (2)
Number of subjects with AEs by system organ class and preferred term								
Blood and lymphatic system disorders	0	0	0	0	0	1 (1)	0	0
Neutropenia	0	0	0	0	0	1 (1)	0	0
Ear and labyrinth disorders	0	0	0	0	0	1 (0)	0	0
Hypoacusis	0	0	0	0	0	1 (0)	0	0
Eye disorders	1 (0)	0	0	0	0	1 (0)	0	0
Conjunctival hyperemia	1 (0)	0	0	0	0	0	0	0
Conjunctival irritation	0	0	0	0	0	1 (0)	0	0
Gastrointestinal disorders	1 (0)	1 (0)	2 (2)	2 (2)	1 (1)	2 (2)	0	0
Abdominal discomfort	1 (0)	0	0	0	0	0	0	0
Abdominal pain upper	0	0	2 (2)	0	1 (1)	0	0	0
Diarrhea	0	0	0	0	0	1 (1)	0	0
Feces hard	0	1 (0)	0	0	0	0	0	0
Feces soft	0	0	0	0	1 (0)	0	0	0
Flatulence	0	0	0	0	0	1 (1)	0	0
Gastroesophageal reflux disease	0	0	0	0	1 (1)	0	0	0
Nausea	0	0	0	2 (2)	1 (1)	0	0	0
Vomiting	0	0	0	0	1 (1)	0	0	0
General disorders and administration site conditions	0	0	0	1 (1)	1 (1)	1 (1)	1 (1)	0
Asthenia	0	0	0	1 (1)	0	0	0	0
Fatigue	0	0	0	0	1 (1)	0	0	0
Feeling abnormal	0	0	0	0	0	0	1 (1)	0
Feeling hot	0	0	0	0	0	1 (1)	0	0
Infections and infestations	1 (1)	0	1 (0)	0	1 (1)	0	0	1 (0)
Folliculitis	0	0	0	0	1 (1)	0	0	0
Hordeolum	0	0	0	0	0	0	0	1 (0)
Upper respiratory tract infection	1 (1)	0	1 (0)	0	0	0	0	0
Injury, poisoning, and procedural complications	1 (0)	0	0	1 (0)	0	0	0	0
Arthropod bite	1 (0)	0	0	1 (0)	0	0	0	0
Metabolism and nutrition disorders	0	0	0	0	1 (1)	0	0	0
Decreased appetite	0	0	0	0	1 (1)	0	0	0
Musculoskeletal and connective tissue disorders	0	1 (0)	1 (0)	0	1 (0)	1 (0)	0	0
Back pain	0	1 (0)	0	0	0	0	0	0
Muscle spasms	0	0	0	0	1 (0)	1 (0)	0	0
Neck pain	0	0	1 (0)	0	0	0	0	0
Nervous system disorders	1 (0)	1 (1)	0	2 (2)	4 (4)	3 (2)	0	0
Dizziness	0	0	0	1 (1)	0	0	0	0
Headache	1 (0)	1 (1)	0	1 (1)	4 (4)	2 (2)	0	0
Presyncope	0	0	0	0	0	1 (0)	0	0
Somnolence	0	0	0	1 (1)	0	0	0	0
Psychiatric disorders	0	0	1 (0)	0	1 (1)	0	0	0
Anxiety	0	0	0	0	1 (1)	0	0	0
Insomnia	0	0	1 (0)	0	0	0	0	0
Renal and urinary disorders	0	0	0	0	1 (0)	0	1 (0)	1 (1)
Nocturia	0	0	0	0	1 (0)	0	0	0
Polyuria	0	0	0	0	0	0	1 (0)	1 (1)
Respiratory, thoracic, and mediastinal disorders	1 (1)	0	0	0	2 (1)	0	1 (1)	0
Epistaxis	0	0	0	0	1 (0)	0	0	0
Nasal congestion	1 (1)	0	0	0	0	0	1 (1)	0
Oropharyngeal pain	0	0	0	0	1 (1)	0	0	0
Skin and subcutaneous tissue disorders	3 (1)	0	0	2 (1)	0	1 (0)	1 (0)	2 (1)
Acne	1 (1)	0	0	1 (1)	0	0	0	1 (1)
Aquagenic pruritus	0	0	0	0	0	1 (0)	0	0
Dermatitis contact	0	0	0	0	0	0	1 (0)	0
Dry skin	1 (0)	0	0	0	0	0	0	0
Ecchymosis	1 (0)	0	0	1 (0)	0	0	0	0
Skin irritation	0	0	0	0	0	0	0	1 (0)

Subjects were counted only once per treatment in each row. The table includes all data collected since the first dose of study drug

*AE* adverse event, *BID* twice daily, *IR* immediate-release, *MAD* multiple ascending doses, *MR* modified-release, *QD* once daily, *QID* four times per day, *TEAE* treatment-emergent AE, *TID* three times per day

^a^Represents placebo groups (IR placebo and MR placebo) in all cohorts

In both studies, all TEAEs were mild or moderate in severity, and most resolved without intervention. There were no dose reductions and/or temporary discontinuations due to TEAEs, serious AEs, or deaths reported in either study. There were no clinically significant changes in vital signs, ECGs, laboratory data, or dose-limiting TEAEs for either study.

In study 2, the presence of asymptomatic, non-adverse, atypical crystals in the urine was identified in 25 subjects (including 3 subjects receiving placebo). The presence of urine crystals at higher abundance (≥ 15/hpf) was restricted to PF-06650833 doses ≥ IR 750 mg BID and was not persistent. None of the subjects presenting with urine crystals had TEAEs, other abnormal clinical signs or symptoms, or clinical laboratory data (serum creatinine or estimated glomerular filtration rate) suggestive of adverse effects on renal function.

### Pharmacokinetics

#### Study 1: SAD

**Fig. 2:**
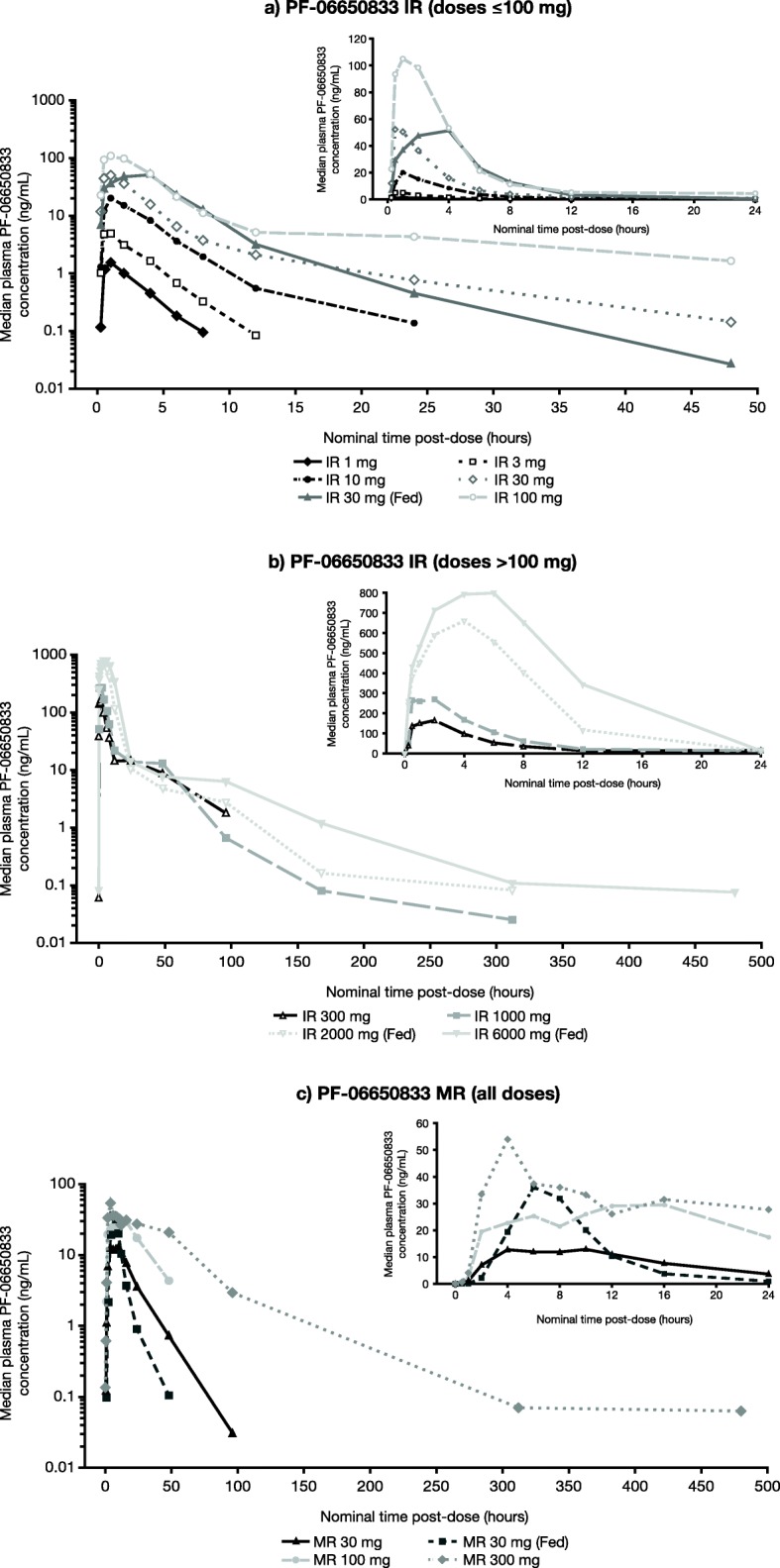
Median plasma concentration-time profile of SAD of PF-06650833 **a** IR doses ≤ 100 mg, **b** IR doses > 100 mg, and **c** MR formulations. All doses were administered orally under fasting conditions (overnight fast of ≥ 10 h) unless otherwise indicated. Fed doses were administered after consumption of a high-fat breakfast meal. Summary statistics were calculated by setting concentration values below the LLOQ to 0. The LLOQ was 0.0500 ng/mL. *IR* immediate-release; *LLOQ* lower limit of quantification; *MR* modified-release; *SAD* single ascending doses

Median plasma concentration-time profiles of SAD of the PF-06650833 IR and MR oral formulations are presented in Fig. 2. Plasma PK parameters for both formulations are summarized in Table 3.

**Table 3:** Plasma PK parameters following SAD of IR and MR PF-06650833 formulations

	IR PF-06650833 dose group													
	IR 1 mg	IR 3 mg	IR 10 mg	IR 30 mg	IR 30 mg (Fed)	IR 100 mg	IR 300 mg	IR 1000 mg	IR 2000 mg (fed)	IR 6000 mg (fed)	MR 30 mg	MR 30 mg (fed)	MR 100 mg	MR 300 mg
*N*, *n*^a^	8, 8	8, 8	8, 7	15, 14	8, 7	8, 5	8, 6	8, 6	8, 0	8, 6	8, 7	8, 8	8, 7	8, 4
AUC_inf_, ng•h/mL	5.141 (37)	17.33 (49)	82.65 (48)	206.4 (29)	325.7 (29)	646.4 (48)	1477 (71)	2429 (40)	NC	10,380 (26)	262.6 (20)	292.7 (19)	838.6 (23)	2042 (45)
AUC_last_, ng•h/mL	4.920 (38)	17.01 (49)	81.91 (44)	203.7 (29)	328.3 (27)	549.4 (38)	1436 (57)	2270 (39)	6346 (26)	10,200 (25)	248.8 (17)	291.6 (19)	762.2 (20)	1968 (39)
*C*_max_, ng/mL	1.747 (22)	5.543 (32)	22.57 (33)	57.96 (34)	53.45 (21)	108.0 (44)	171.7 (46)	308.8 (34)	650.0 (27)	847.2 (21)	15.76 (33)	41.24 (34)	42.81 (36)	64.51 (46)
*T*_max_, h^b^	1 (0.500–1.00)	0.525 (0.500–1.00)	1 (0.500–4.00)	0.517 (0.500–2.00)	4.00 (2.00–6.00)	1 (0.500–2.03)	2 (0.500–2.00)	0.75 (0.500–2.00)	4 (2.00–6.00)	6 (2.00–6.15)	6.00 (4.00–12.2)	6.00 (4.00–8.02)	8.00 (2.00–16.0)	4.00 (2.00–12.0)
*t*_½_, h^c^	1.86 ± 0.240	2.34 ± 0.670	3.54 ± 0.139	10.2 ± 6.06	4.43 ± 1.43	15.0 ± 5.22	19.9 ± 9.54	44.9 ± 69.6	NC	72.1 ± 53.9	11.7 ± 3.26	6.33 ± 1.66	9.35 ± 3.62	38.8 ± 21.1
CL/F, L/h	194.3 (37)	173.1 (49)	121.0 (48)	145.5 (29)	92.08 (29)	154.7 (48)	203.3 (71)	411.4 (40)	NC	577.8 (26)	114.1 (20)	102.5 (19)	119.0 (23)	147.1 (45)
*V*_*z*_/*F*, L	516.9 (31)	567.3 (39)	617.5 (50)	1829 (80)	565.5 (27)	3189 (51)	5310 (69)	14,600 (163)	NC	49,220 (71)	1865 (35)	898.6 (33)	1476 (31)	6817 (46)
*C*_last_, ng/mL	0.08150 (25)	0.09984 (69)	0.1366 (54)	0.2196 (84)	0.1651 (98)	1.218 (147)	0.9585 (411)	0.07431 (48)	0.1599 (348)	0.08257 (35)	0.2631 (233)	0.1490 (91)	1.881 (345)	0.1478 (435)
*T*_last_, h^b^	8.04 (8.00–12.0)	12 (12.0–24.0)	24 (24.0–24.0)	48 (24.0–48.1)	36.0 (24.0–48.0)	48 (48.0–48.0)	96 (96.0–96.0)	170 (168–363)	313 (169–313)	480 (192–481)	72.0 (48.0–96.0)	72.0 (48.0–96.0)	48.0 (48.0–48.0)^d^	493 (96.0–528)
MRT, h^c^	3.12 ± 0.525	3.40 ± 0.622	4.31 ± 0.787	6.82 ± 3.38	5.43 ± 1.10	11.2 ± 4.81	20.6 ± 12.6	18.7 ± 4.37	NC	18.1 ± 6.11	17.5 ± 2.35	9.27 ± 0.60	19.3 ± 5.73	50.6 ± 15.9

Data presented as geometric mean (% geometric coefficient of variation) unless otherwise noted. AUC_inf_ and *t*_½_ were reported for all treatments when the following criteria were met: a well-characterized terminal phase defined as one with at least three data points and a goodness-of-fit statistic for the log-linear regression (*r*^2^) ≥ 0.9. In addition, AUC_inf_ was reported since the percentage of AUC extrapolated from AUC_last_ was < 20% for all subjects

All doses were administered orally under fasting conditions (overnight fast of ≥ 10 h) unless otherwise indicated. Fed doses were administered after consumption of a high-fat breakfast meal

*AUC* area under the concentration-time profile curve, *AUC*_*inf*_ AUC from time zero extrapolated to infinity, *AUC*_*last*_ AUC from *C*_last_, *CL/F* apparent oral clearance, *C*_*last*_ last quantifiable concentration, *C*_*max*_ maximum observed concentration, *IR* immediate-release, *MR* modified-release, *MRT* mean residence time, *NC* not calculated if fewer than three subjects had reportable parameter values, *PK* pharmacokinetic, *SAD* single ascending doses, *t*_*½*_ terminal half-life, *T*_*last*_ time of C_last_, *T*_*max*_ time of C_max_, *V*_*z*_*/F* apparent volume of distribution

^a^*N*, number of evaluable subjects; *n*, number of subjects where *t*_*½*_, AUC_inf_, CL/F, *V*_*z*_/*F*, and MRT were determined

^b^Median (range)

^c^Mean (± standard deviation)

^d^Data were only received for MR 100 mg up to 48 h (instead of 96 h); thus, data are not presented for PF-06650833 at 96 h and *T*_last_ was 48.0 h

When administered in the fasted state, plasma concentrations of PF-06650833 increased in a dose-dependent manner with SAD of ≤ 100 mg for both IR and MR formulations and in a less than proportional manner with higher doses; lower *C*_max_ values were observed for all comparable doses of MR versus IR formulations as expected. Dose-normalized *C*_max_ and AUC from time zero extrapolated to infinity (AUC_inf_) values for PF-06650833 IR and MR formulations are shown in Additional file 1: Figure S1. Absorption was rapid for the PF-06650833 IR formulation (median time of *C*_max_ [*T*_max_] 0.5–2.0 h across the 1–1000 mg dose range) compared with the more gradual absorption of the MR formulation (median *T*_max_ 4.0–8.0 h over the 30–300 mg dose range). Half-life was similar for PF-06650833 IR versus MR formulations at comparable nominal doses of 30–100 mg (mean terminal half-life [*t*_½_] 10.2–15.0 and 9.4–11.7 h, respectively) but was longer for MR 300 mg (mean *t*_½_ 38.8 h) versus IR 300 mg (mean *t*_½_ 19.9 h), for which a longer terminal phase was measured.

Following single oral doses of PF-06650833 IR and MR 30 mg formulations, absorption was delayed in the fed (high-fat meal) versus fasted (≥ 10 h) state for the IR 30 mg dose (median *T*_max_ 4.0 versus 0.5 h), but was not affected by food for the MR 30 mg dose (median *T*_max_ 6.0 h for both). Half-life was reduced in the fed state for both IR (*t*_½_ 10.2 to 4.4 h) and MR (*t*_½_ 11.7 to 6.33 h) formulations.

Co-administration of IR 30 mg with a high-fat meal increased total exposure by 33% (AUC_inf_ [ng•h/mL] 211.3 versus 316.1 for fasted and fed conditions, respectively; fasted/fed ratio 66.8% [95% CI 57.49, 77.71]), but did not have an effect on *C*_max_ (57.62 versus 54.21 ng/mL for fasted and fed conditions, respectively; fasted/fed ratio 106.30% [95% CI 83.69, 135.03]). Conversely, *C*_max_ increased by 62% in the fed state for MR 30 mg (*C*_max_ 14.97 versus 39.34 ng/mL for fasted and fed conditions, respectively; fasted/fed ratio 38.05% [95% CI 29.45, 49.16]), while total exposure remained unaffected (AUC_inf_ 260.9 versus 287.1 ng h/mL for fasted and fed conditions, respectively; fasted/fed ratio 90.86% [95% CI 78.40, 105.29]).

When 2000 and 6000 mg IR doses were administered in the fed state, median *T*_max_ was 4.0 and 6.0 h, respectively. Following the attainment of *C*_max_, PF-06650833 concentrations demonstrated a multiphasic decline. Mean *t*_½_ was 72.1 h for PF-06650833 IR 6000 mg (and was not able to be determined for IR 2000 mg). In general, the increase in exposure was less than proportional to the increase in dose for the 2000 and 6000 mg IR formulations compared with the lower dose groups.

#### Study 2: MAD

**Fig. 3:**
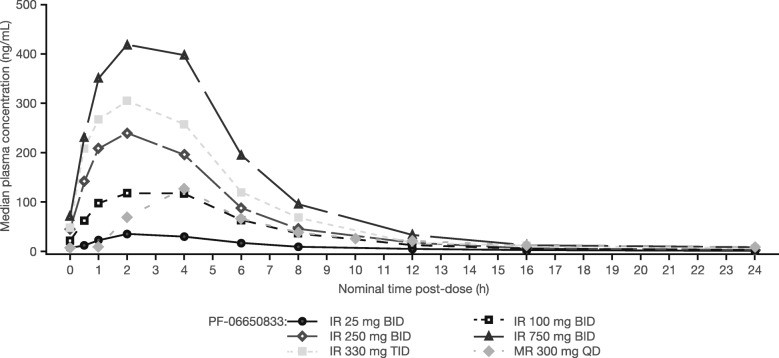
Median plasma concentration-time profile of MAD of PF-06650833 IR and MR formulations at steady state on day 14. Time post-dose refers to the first morning dose on day 14. Day 14 data for cohort 5 (IR 1000 mg QID) were not available due to premature discontinuation of this cohort on day 9. Summary statistics were calculated by setting concentration values BLQ to 0. The LLOQ was 0.0500 ng/mL, except four pre-dose samples with LLOQ of 0.100 ng/mL. All doses were administered orally under fed conditions (standard meal). *BID* twice daily; *BLQ* below lower limit of quantification; *IR* immediate-release; *LLOQ* lower limit of quantification; *MAD* multiple ascending doses; *MR* modified-release; *QD* once daily; *QID* four times per day; *TID* three times per day

Median plasma concentration-time profiles at steady state on day 14 administration of oral MAD of PF-06650833 IR and MR formulations are presented in Fig. 3. Plasma and urine PK parameters for both formulations on day 1 and day 14 are summarized in Table 4.

**Table 4:** Plasma and urine PK parameters following MAD of IR and MR PF-06650833

	PF-06650833 dose group						
	IR 25 mg BID	IR 100 mg BID	IR 250 mg BID	IR 750 mg BID	IR 1000 mg QID^a^	IR 330 mg TID	MR 300 mg QD
Day 1							
*N*	8	8	8	8	8	8	8
AUC_tau_, ng•h/mL	163.7 (26)	566.3 (32)	1348 (40)	2581 (28)	2008 (25)	1500 (38)	1150 (62)
*C*_max_, ng/mL	29.96 (34)	101.8 (38)	226.4 (35)	470.3 (24)	517.3 (30)	309.0 (37)	149.3 (51)
*T*_max_, h^b^	2.03 (1.00–4.00)	2.03 (1.00–4.00)	4.00 (2.00–4.00)	3.12 (2.03–4.07)	4.00 (2.00–4.02)	2.03 (2.00–4.00)	4.00 (2.00–10.0)
Day 14							
*N*	8	8	8	7	–	8	7
AUC_tau_, ng•h/mL	204.2 (26)	776.5 (16)	1360 (39)	2475 (24)	–	1599 (28)	975.4 (65)
*C*_max_, ng/mL	36.19 (31)	127.6 (16)	238.5 (33)	420.7 (25)	–	317.9 (27)	146.8 (49)
*T*_max_, h^b^	2.00 (0.500–4.00)	2.02 (1.00–4.00)	2.02 (2.00–4.03)	2.00 (2.00–4.00)	–	2.00 (1.00–4.00)	4.00 (2.00–7.50)
CL/F, L/h	122.4 (26)	128.9 (16)	183.7 (39)	302.9 (24)	–	206.1 (28)	308.0 (65)
*V*_*z*_/*F*, L	9650 (37)	8064 (27)	10,170 (44)	13,160 (29)	–	10,070 (24)	11,600 (46)
*C*_min_, ng/mL	4.064 (46)	14.13 (40)	20.07 (76)	33.07 (50)	–	51.48 (68)	6.471 (55)
*C*_av_, ng/mL	17.03 (26)	64.72 (16)	113.3 (39)	206.3 (24)	–	200.1 (28)	40.62 (65)
PTF	1.868 (21)	1.740 (16)	1.911 (14)	1.868 (12)	–	1.307 (18)	3.383 (37)
*R*_ac_	1.250 (19)	1.373 (23)	1.009 (13)	0.9377 (32)	–	1.068 (26)	0.8985 (24)
*R*_ac_, _Cmax_	1.209 (22)	1.253 (28)	1.052 (22)	0.8689 (38)	–	1.029 (28)	1.078 (29)
*t*_½_, h^c^	NR	NR	NR	29.4^d^ ± 1.78	–	31.4^e^ ± 5.60	25.4^f^ ± 6.41
MRT, h	NR	NR	NR	6.15^d^ ± 0.366	–	5.29^e^ ± 1.18	20.3^f^ ± 9.46
A_e24_%	0.7288 (27)	0.9414 (29)	0.4822 (53)	0.4885 (24)	–	0.6372 (34)	0.300 (70)
CL_r_, mL/min	13.62 (19)	18.54 (19)	13.92 (18)	23.14 (19)	–	18.83 (21)	15.39 (30)

Data presented as geometric mean (% geometric coefficient of variation) unless otherwise noted

All doses were administered orally under fed conditions (standard meal)

*A*_*e24*_ cumulative amount of drug recovered unchanged in urine up to 24 h; *AUC* area under the concentration-time profile curve; *AUC*_*tau*_ AUC from time 0 to time tau, the dosing interval, where tau = 6, 8, 12, and 24 h for QID, TID, BID, and QD dosing, respectively; *BID* twice daily; *C*_*av*_ average concentration for the dosing interval; *CL/F* apparent oral clearance; *CL*_*r*_ renal clearance; *C*_*max*_ maximum observed concentration; *C*_*min*_ lowest concentration observed during the dosing interval; *IR* immediate-release; *MAD* multiple ascending doses; *MR* modified-release; *MRT* mean residence time; *NR* not recorded; *PK* pharmacokinetic; *PTF* peak-trough fluctuation; *QD* once daily; *QID* four times per day; *R*_*ac*_ observed accumulation ratio; *t*_*½*_ terminal half-life; *TID* three times per day; *T*_*max*_ time of C_max_; *V*_*z*_*/F* apparent volume of distribution

^a^Day 14 data for cohort 5 (PF-06650833 IR 1000 mg QID) were not available due to discontinuation of this cohort on day 9

^b^Median (range)

^c^Mean (± standard deviation)

^d^*N* = 4

^e^*N* = 3

^f^*N* = 6

The PF-06650833 absorption rate was slightly faster on day 1 following initial oral doses of IR 25 to 1000 mg under fed (standard meal) conditions (median *T*_max_ 2–4 h), compared with the more gradual absorption of the MR formulation (median *T*_max_ 4 h). On day 14, absorption rates were comparable with day 1 values for PF-06650833 IR (median *T*_max_ 2 h) and MR (median *T*_max_ 4 h) formulations.

Steady state was reached by day 4 for all PF-06650833 dose groups. Across all dosages, mean oral clearance values ranged from 122.4 to 308.0 L/h, and mean volume of distribution values ranged from 8064 L to 13,160 L. Mean half-life values calculated for IR 750 mg BID, IR 330 mg TID, and MR 300 mg QD dosages ranged from 25.4 to 31.4 h. On day 14, AUC from time 0 to time tau (AUC_tau_; where tau is the dosing interval [6, 8, 12, and 24 h for QID, TID, BID, and QD dosing, respectively]) and *C*_max_ increased proportionally for IR 25 to 100 mg BID doses, with less than proportional increases observed at doses ≥ 250 mg.

Accumulation ranged from 0.9-fold to 1.4-fold for AUC_tau_ and 0.9-fold to 1.3-fold for *C*_max_. Less than 1% of the dose was recovered unchanged in the urine for all dose groups, with renal clearance ranging from 14 to 19 mL/min for IR 25 mg BID, IR 330 mg TID, and MR 300 mg QD, and 23 mL/min for IR 750 mg BID. Dose-normalized *C*_max_ and AUC_tau_ following MAD of PF-06650833 IR and MR formulations on day 14 are shown in Additional file 1: Figure S2.

Ratios of 4β-hydroxycholesterol to cholesterol were comparable (< 20% mean change) between day 14 (4 h post-dose) and day 1 (pre-dose) across the dose groups for doses up to 750 mg BID, indicating no apparent trend for CYP3A induction or inhibition [21].

### Pharmacodynamics

#### Study 2: MAD

**Fig. 4:**
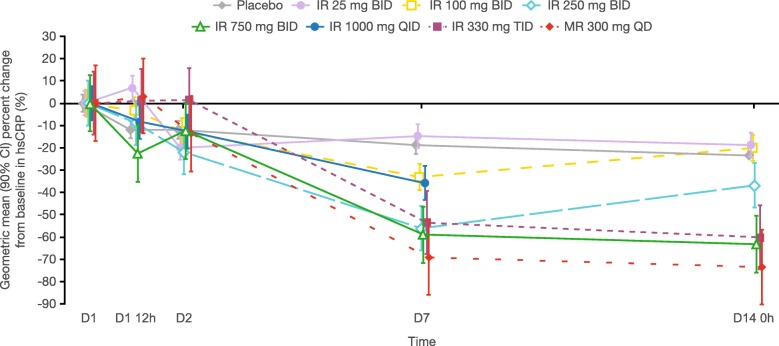
Geometric mean (90% CI) change from baseline in serum hsCRP following MAD of IR and MR PF-06650833 formulations. Baseline was defined as the last pre-dose measurement taken on day 1. Time post-dose refers to the first morning dose. Values below the LLOQ were set to half of the LLOQ in the calculation. The LLOQ of hsCRP was 0.015 mg/dL. Unplanned readings and early withdrawal readings are excluded. The dosing in 1000 mg QID dose group was stopped by the sponsor after the second dose on day 9, and the subjects had their follow-up visits 2 days (approximately 43 h) and 13 days (approximately 310 h) after the last dose on day 9. *BID* twice daily; *CI* confidence interval; *D* day; *h* hour; *hsCRP* high-sensitivity C-reactive protein; *IR* immediate-release; *LLOQ* lower limit of quantification; *MAD* multiple ascending doses

Geometric mean serum hsCRP levels ranged from 0.067 to 0.101 mg/dL across the dose groups at baseline. There was a sustained decrease from baseline in serum hsCRP, which, in general, reached maximal reduction by day 7, following administration of PF-06650833 IR formulations ≥ 250 mg BID and in the MR 300 mg QD dose group (Fig. 4). On day 14, reductions from baseline in hsCRP of approximately 60–70% (geometric mean percentage) were seen in the highest dose groups.

## Discussion

These phase 1 studies evaluated the safety, tolerability, PK, and PD of SAD and MAD of IR and MR formulations of PF-06650833, an IRAK4 inhibitor, in healthy adult subjects. PF-06650833 has a favorable safety profile and was well tolerated in single oral doses of IR formulations up to 6000 mg (with food [high-fat meal]) and oral MR formulations up to 300 mg, and in multiple oral IR doses up to 1000 mg QID and MR doses up to 300 mg QD (all with standard meal), with no dose-limiting adverse effects identified.

In both studies, the most common treatment-related TEAEs included headache, gastrointestinal disorders, and acne; there were no clinically significant changes in laboratory test results or vital sign parameters at any dose.

In the SAD study, the frequency of TEAEs was slightly more common with PF-06650833 IR doses ≥ 1000 mg. In the MAD study, the highest number of TEAEs occurred in the IR 750 mg BID dose group (the second highest dose tested). TEAEs in both studies were all mild to moderate in severity, and most resolved without intervention. One subject in the IR 1000 mg QID dose group of study 2 was discontinued prematurely for neutropenia having met a protocol-defined ANC threshold for discontinuation. The subject had borderline low ANC at baseline and was asymptomatic. Review of historical laboratory findings for this subject suggested that the subject may have had previously undiagnosed cyclic benign ethnic neutropenia, which could have accounted for the observed fluctuation in ANC, although a causal relationship to PF-06650833 cannot be formally excluded. There were no dose reductions/temporary discontinuations due to TEAEs, serious AEs, or deaths reported in either study. Definitive conclusions about the frequency of TEAEs relative to placebo are limited by the small sample size and relative paucity of TEAEs, and clear dose or regimen relationships were not established.

In the MAD study, atypical crystals were detected in the urine of a total of 22 subjects receiving total daily IR doses ≥ 330 mg TID or MR 300 mg of PF-06650833 and in 3 subjects receiving placebo. The crystals were found in the urinalyses performed as part of a routine safety laboratory monitoring. The atypical urine crystals were not consistently observed in repeat visits for an individual subject nor was there a pattern observed between subjects at a given dose. Urine crystals at higher abundance (≥ 15/hpf) were only observed at the highest 2 doses (≥ IR 750 mg BID), which are in excess of those that are likely to be explored in future clinical trials of PF-06650833. The manifestation of the atypical urine crystals was entirely asymptomatic, was not associated with any clinical or laboratory evidence of adverse effects on the kidney, and was therefore not considered adverse. The etiology of the atypical crystals is currently uncertain but may represent precipitation of parent drug and/or metabolites in the urine under supersaturated conditions. As the atypical urine crystals were not accompanied by any renal findings, their clinical significance, if any, is unclear, and their occurrence is not an impediment to further clinical development of PF-06650833 for the treatment of autoimmune and inflammatory diseases.

For single oral doses of IR 1–1000 mg in the fasted state in the SAD study, the increase of PF-06650833 exposure was dose linear over the 1–100 mg dose range and less than dose linear at higher doses. Consistent with being a high-permeability compound [16], the absorption of PF-06650833 in the fasted state was rapid for the IR formulation, with a median *T*_max_ ranging from 0.5 to 2 h across the dose groups. The apparent half-life of PF-06650833 appeared to increase with dose, with a mean half-life of 1.86 h at 1 mg and of 44.9 h at 1000 mg, but with large variability. The much shorter half-life observed at lower doses could be due to the serum concentrations of PF-06650833 falling below the quantifiable limit before reaching the terminal elimination phase, which began at approximately 12 h after dosing. The longer and more variable half-life observed at higher doses could possibly be due to the flip-flop kinetics at the high-dose levels. In addition, multiple peaks were observed in concentration-time profiles of a few subjects after administration of the IR formulation, indicating the possibility of a recycling mechanism, which may also have contributed to a longer terminal elimination phase. PK studies including intravenous dosing would be required to fully delineate the PK of PF-06650833.

MR formulations were developed in order to decrease the dosing frequency and lower the peak-to-trough ratio in future trials. The safety and tolerability of these MR formulations were evaluated in both SAD and MAD studies. Consistent with the general properties of MR formulations, the MR formulation demonstrated delayed *T*_max_ and tapered *C*_max_ in fasted states. For example, at 30 mg, the median *T*_max_ was 0.5 h and *C*_max_ was 58 ng/mL for the IR formulation, while median *T*_max_ was 6 h and *C*_max_ was 15.8 ng/mL with the MR formulation at the same dose. In the fasted state, AUC_inf_ of the MR formulation was also slightly higher compared with the IR formulation at an equivalent dose. In general, in the fasted state, the MR formulation displayed relatively flat concentration-time profiles during the long absorption phase. Overall, the MR formulation exhibited PK characteristics in the fasted state suitable for QD dosing.

Single dose administration of IR formulations of PF-06650833 with a high-fat meal delayed oral absorption of the PF-06650833 IR 30 mg formulation (median *T*_max_ of 0.5 and 4 h under fasted and fed states, respectively) and increased total exposure by 33% without affecting the *C*_max_. In contrast, high-fat meal intake did not delay the absorption of the MR 30 mg dose (median *T*_max_ of 6 h in both fasted and fed states), increased *C*_max_ by more than twofold, and decreased *t*_½_ by twofold. The shift in *T*_max_ and the increase in AUC for the IR formulation in the fed state could be due to an increase in gastric residence time and possible absorption from the upper part of the gastrointestinal tract, whereas the absorption of the MR formulation may be occurring predominantly in the lower part of the gastrointestinal tract.

In the MAD study, on day 1, following single doses of IR 25 to 1000 mg and MR 300 mg, the standard meal led to a slightly more modest delay of absorption compared with the high-fat meal in the SAD study. For example, the median *T*_max_ of IR 25 mg under standard meal conditions was 2 h, while the median *T*_max_ of IR 30 mg in the SAD study was 0.5 and 4 h under fasted and high-fat meal states, respectively. The MR formulation maintained a long absorption phase, with a median *T*_max_ of 4 h and individual *T*_max_ ranging from 2 to 10 h for the MR 300 mg dose under standard meal conditions. At lower doses, half-lives were not reportable either due to *r*^2^ < 0.9 or the proportion of extrapolation of AUC being higher than 20%. The half-life for 1000 mg QID was not determined, because the cohort was not dosed beyond day 10 and plasma samples were not collected. The *t*_½_ of PF-06650833 at steady state ranged between 25 and 31 h for the dose groups with reportable half-lives. The accumulation of PF-06650833 for the various dose groups was low, and steady state was reached by day 4 for all dose groups based on the evaluation of *C*_min._

There was a sustained decrease from baseline in serum hsCRP from day 7 to day 14 following administration of multiple PF-06650833 IR formulations with a total daily dose of ≥ 250 mg BID and in the MR 300 mg QD dose group. Since hsCRP is a general marker of inflammation, and specifically a marker of disease activity in RA and other inflammatory diseases, seeing an effect in healthy subjects is encouraging and consistent with PF-06650833 having a pharmacologic effect downstream in the TLR signaling cascade (and likely upstream of IL-6). In concert with prior in vitro and in vivo studies [16–18] and available preclinical data [16], these results suggest that PF-06650833 may have clinically relevant anti-inflammatory effects, supporting its development for the treatment of autoimmune diseases.

## Conclusions

PF-06650833, the first IRAK4 inhibitor to enter human clinical trials, was shown to have a favorable safety profile and be well tolerated in healthy adult subjects up to a single dose of IR 6000 mg (with food [high-fat meal]) and multiple doses up to IR 1000 mg QID and MR 300 mg QD (with food [standard meal]), with no dose-limiting adverse effects observed. PK data demonstrated generally anticipated effects on exposure with increasing dose and the effect of food, for both IR and MR formulations. The MR formulation provided sustained exposures that have the potential to allow QD dosing. The accumulated data support continued evaluation in human clinical trials for the treatment of rheumatic and other autoimmune diseases.

## Supplementary information

**Additional file 1: Supplemental Methods.** Randomization. Blood and urine collection for PK/PD analyses. Blood and urine collection for analysis of safety laboratory parameters. Analysis of vital signs. **Figure S1.** Dose-normalized a) C_max_ and b) AUC_inf_ following SAD of IR and MR PF-06650833 formulations. **Figure S2.** Dose-normalized a) C_max_ and b) AUC_tau_ (day 14) following MAD of IR and MR PF-06650833 formulations.

## Data Availability

Upon request, and subject to certain criteria, conditions, and exceptions (see https://www.pfizer.com/science/clinical-trials/trial-data-and-results for more information), Pfizer will provide access to individual de-identified participant data from Pfizer-sponsored global interventional clinical studies conducted for medicines, vaccines, and medical devices (1) for indications that have been approved in the USA and/or EU or (2) in programs that have been terminated (i.e., development for all indications has been discontinued). Pfizer will also consider requests for the protocol, data dictionary, and statistical analysis plan. Data may be requested from Pfizer trials 24 months after study completion. The de-identified participant data will be made available to researchers whose proposals meet the research criteria and other conditions and, for which an exception does not apply, via a secure portal. To gain access, data requestors must enter into a data access agreement with Pfizer.

## Abbreviations

AE: Adverse event
A_e24_: Cumulative amount of drug recovered unchanged in urine up to 24 h
ANC: Absolute neutrophil count
AUC: Area under the concentration-time profile curve
AUC_inf_: AUC from time zero extrapolated to infinity
AUC_last_: AUC from *C*_last_
AUC_tau_: AUC from time 0 to time tau, the dosing interval, where tau = 6, 8, 12, and 24 h for QID, TID, BID, and QD dosing, respectively
BID: Twice daily
BLQ: Below lower limit of quantification
BMI: Body mass index
*C*_av_: Average concentration for the dosing interval
CI: Confidence interval
CL/F: Apparent oral clearance
*C*_last_: Last quantifiable concentration
CL_*r*_: Renal clearance
*C*_max_: Maximum observed concentration
*C*_min_: Lowest concentration observed during the dosing interval
CYP3A: Cytochrome P450 3A
CYP3A4: Cytochrome P450 3A4
Dn: Dose-normalized
ECG: Electrocardiogram
hpf: High power field
hsCRP: High-sensitivity C-reactive protein
IL: Interleukin
IR: Immediate-release
IRAK4: Interleukin-1 receptor-associated kinase 4
LC-MS: Liquid chromatography tandem mass spectrometric methods
LLOQ: Lower limit of quantification
MAD: Multiple ascending dose
MR: Modified-release
MRT: Mean residence time
MTD: Maximum tolerated dose
NC: Not calculated
NOAEL: No observable adverse effect level
NR: Not recorded
PD: Pharmacodynamics
PK: Pharmacokinetic
PTF: Peak-trough fluctuation
QD: Once daily
QID: Four times per day
RA: Rheumatoid arthritis
*R*_ac_: Observed accumulation ratio
SAD: Single ascending dose
SD: Standard deviation
SLE: Systemic lupus erythematosus
*t*_*½*_: Terminal half-life
TEAE: Treatment-emergent adverse event
TID: Three times per day
TK: Toxicokinetic limit
*T*_last_: Time of *C*_last_
TLR: Toll-like receptor
*T*_max_: Time of *C*_max_
*V*_*z*_/*F*: Apparent volume of distribution
