# Supplementary material for: Safety, tolerability, pharmacokinetics, and pharmacodynamics of PF-06650833, a selective interleukin-1 receptor-associated kinase 4 (IRAK4) inhibitor, in single and multiple ascending dose randomized phase 1 studies in healthy subjects
Source: Arthritis Res Ther. 2019 Dec 5;21:269. doi: 10.1186/s13075-019-2008-6 (PMC6896740; doi:10.1186/s13075-019-2008-6)
Supplement: Supplementary file 1 — Additional file 1: Supplemental Methods. Randomization. Blood and urine collection for PK/PD analyses. Blood and urine collection for analysis of safety laboratory parameters. Analysis of vital signs. Figure S1. Dose-normalized a) Cmax and b) AUCinf following SAD of IR and MR PF-06650833 formulations. Figure S2. Dose-normalized a) Cmax and b) AUCtau (day 14) following MAD of IR and MR PF-06650833 formulations. [file 13075_2019_2008_MOESM1_ESM.pdf]

Additional file 1

Supplemental Methods

Randomization

In both studies, subjects were assigned to dose groups according to a pre-defined randomization schedule. Each subject was given a unique Electronic Data Capture Management System identification entry. Prior to dosing, a randomization number corresponding to a treatment schedule determined by a study sponsor-generated randomization code was allocated to each subject and retained throughout the study. This number also appeared on the dispensing containers for the study treatments.

Blood and urine collection for PK/PD analyses

In Study 1, blood samples were collected for PK analysis on day 0 at 0 hours (prior to dosing with PF-06650833/placebo), 0.25, 0.5, 1, 2, 4, 6, 8, 10, 12, and 16 hours post-dose; day 2 at 24 hours post-dose; day 3 at 48 hours post-dose; day 5 at 96 hours post-dose; and at follow-up on days 8 (168 hours), 14 (312 hours), and 21 (480 hours). Samples were collected before breakfast when dosing was done in the fasted state. Subjects administered the modified-release formulation had additional PK blood sample collection on day 1 at 10 and 16 hours post-dose, and did not have a 0.25-hour PK blood sample collection. In response to emerging PK data, additional PK sample collections were obtained as follows: day 5 (96 hours post-dose) for all periods beginning with cohort 2, period 4; day 8 (168 hours post-dose) to the final period of each cohort beginning with cohort 3; day 14 (312 hours post-dose); and day 21 (480 hours post-dose) of the final periods of cohorts 2 through 4.

In Study 2, pre-dose blood samples were collected for PK analysis on days 1, 2, 4, 7, 10, and 14. Post-dose samples were collected for PK analysis on days 1 and 14 at 0.5, 1, 2, 4, 6, 8, 10, 12, and 16 hours post-dose; on day 7 at 1, 2, 4, 6, 8, and 12 hours post-dose; and on days 15, 16, 18, and 22 at 24, 48, 96, and 192 hours post-dose relative to day 14 administration, respectively. Urine was collected on days 1 and 14 at 0.5, 1, 2, 4, 6, 8, 10, 12, 16, and 24 hours post-dose. Blood samples were collected for PD analysis prior to morning dosing on dosing days and before breakfast on non-dosing days on days 0, 1, 2, 7, 14, 15, and 22. 4β-hydroxycholesterol/cholesterol ratio was collected on day 1 at pre-dose (0 hours) only. 4β-hydroxycholesterol/cholesterol was collected on day 14 and day 22 at 4 hours post-dosing only.

Blood samples (2 mL, to obtain ≥0.5 mL plasma) were collected in K_2EDTA tubes (dipotassium ethylenediaminetetraacetic acid) and centrifuged at ~1700 x g for 10 minutes at 4°C, with plasma then stored in polypropylene tubes at -70°C within 1 hour of collection. Plasma samples were analyzed using a validated, sensitive, and specific liquid chromatography tandem mass spectrometric method (LC-MS; at Pfizer Inc, Groton, CT, USA for Study 1; and at Worldwide Pharmacokinetics, Dynamics, and Metabolism, Cambridge, MA, USA for Study 2). The lower limit of quantification (LLOQ) for PF-06650833 in plasma was 0.0500 ng/mL. Between-day assay accuracy values were also calculated (not shown).

In Study 2 only, urine samples were collected in aliquots pre-dose. Cumulative 0–12-hour and 12–24-hour urine samples were also collected as follows: from day 1 after morning dosing to day 2 before morning dosing (and before breakfast) for cohorts 6–8 only; and from day 14 after morning dosing to day 15 before morning dosing (and before breakfast) for all cohorts. Urine samples were also analyzed using a validated, sensitive, and specific LC-MS method (Worldwide Pharmacokinetics, Dynamics, and Metabolism, Cambridge, MA, USA).

The LLOQ for PF-06650833 in urine was 1.00 ng/mL. Between-day assay accuracy values were calculated (not shown). Urine samples were also retained for metabolite analyses.

Blood samples (3.5 mL, to obtain ~1.2 mL serum) for analysis of the exploratory biomarker high-sensitivity C-reactive protein (hsCRP) were collected on days 0, 1, 2, 7, 14, 15, and 22. Serum samples were analyzed for hsCRP as part of routine clinical laboratory assessments.

Blood samples (3 mL) to provide approximately 1.2 mL plasma for the analysis of 4β-hydroxycholesterol and cholesterol were collected into appropriately labeled tubes containing lithium heparin. Blood samples were analyzed for 4β-hydroxycholesterol and cholesterol using a validated liquid chromatography tandem mass spectrometric method.

Blood and urine collection for analysis of safety laboratory parameters

In Study 1, blood samples were collected for analysis of safety (hematology and chemistry) at screening and on days 0, 2, 3, 5, and at follow-up on days 8, 14, and 21. Urine was collected for urinalysis at screening and on days 0, 2, 3, 5, and at follow-up on day 8. In Study 2, blood samples were collected for analysis of safety (hematology and chemistry) and urine was collected for urinalysis at screening and on days 0, 1, 4, 7, 10, 14, 18, and 22.

Analysis of vital signs

In Study 1, supine blood pressure was measured on days 0, 3, 5, and at follow-up on day 8. ECGs were conducted at screening and on days 0, 2, 5, and at follow-up on days 8, 14 (cohort 4, period 2 only), and 21. In Study 2, supine pulse, blood pressure, and ECG were measured at screening and on days 1, 4, 7, 10, 14, 15, 18, and 22.

Fig. S1 Dose-normalized **a)** C_{max} and **b)** AUC_{inf} following SAD of IR and MR PF-06650833 formulations

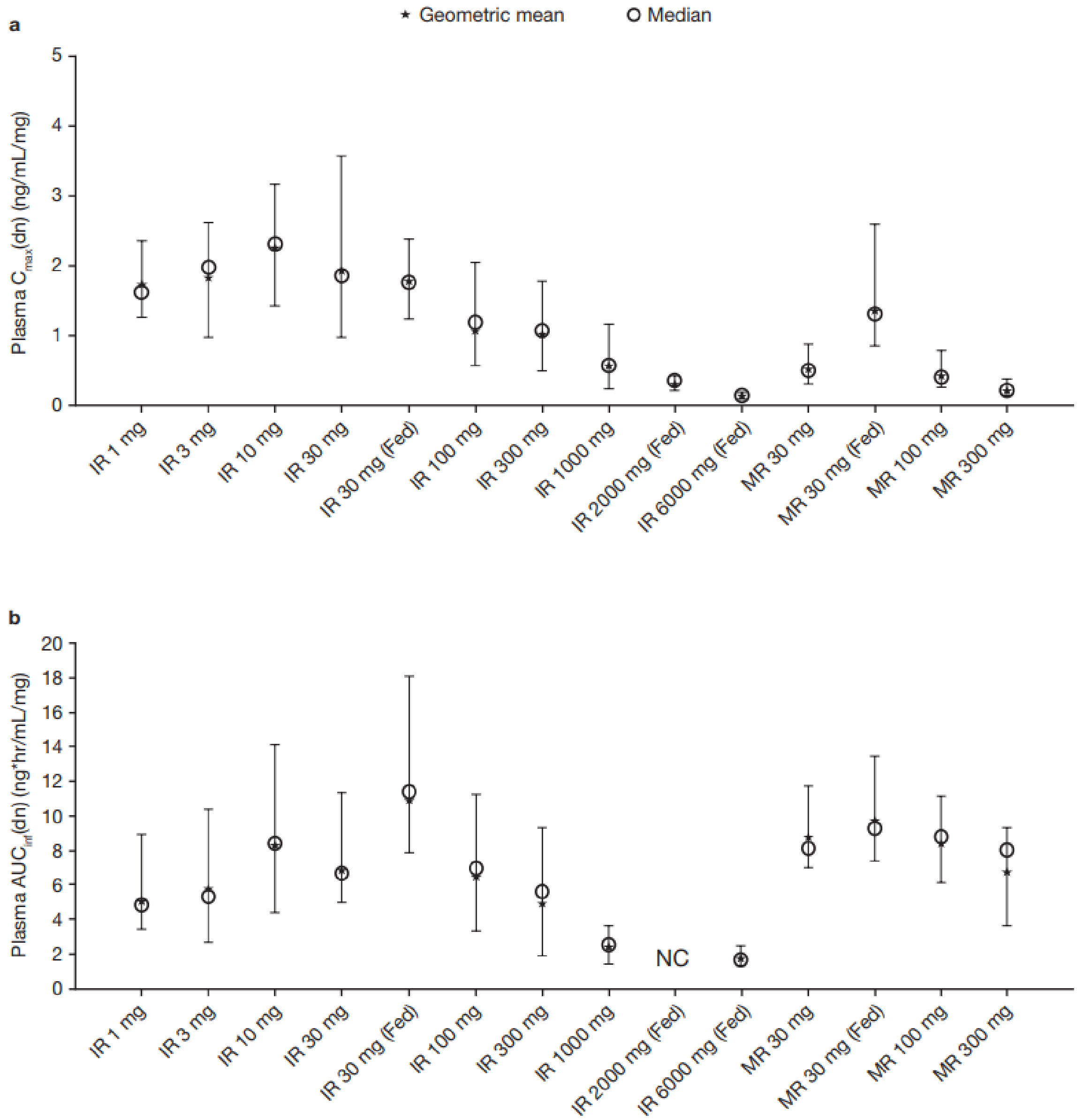

Dose normalized to a 1 mg dose

The geometric mean is calculated for each dose group; stars represent geometric means Bars represent the range of minimum and maximum values

All doses were administered orally under fasting conditions (overnight fast of ≥10 hours) unless otherwise indicated. Fed doses were administered after consumption of a high-fat breakfast meal

AUC area under the curve, AUC_{inf} AUC from time zero extrapolated to infinity, C_{max} maximum observed concentration, *dn* dose-normalized, *IR* immediate-release, *MR* modified-release, *NC* not calculated, *SAD* single ascending doses

Fig. S2 Dose-normalized **a)** C_{max} and **b)** AUC_{tau} (day 14) following MAD of IR and MR PF-06650833 formulations

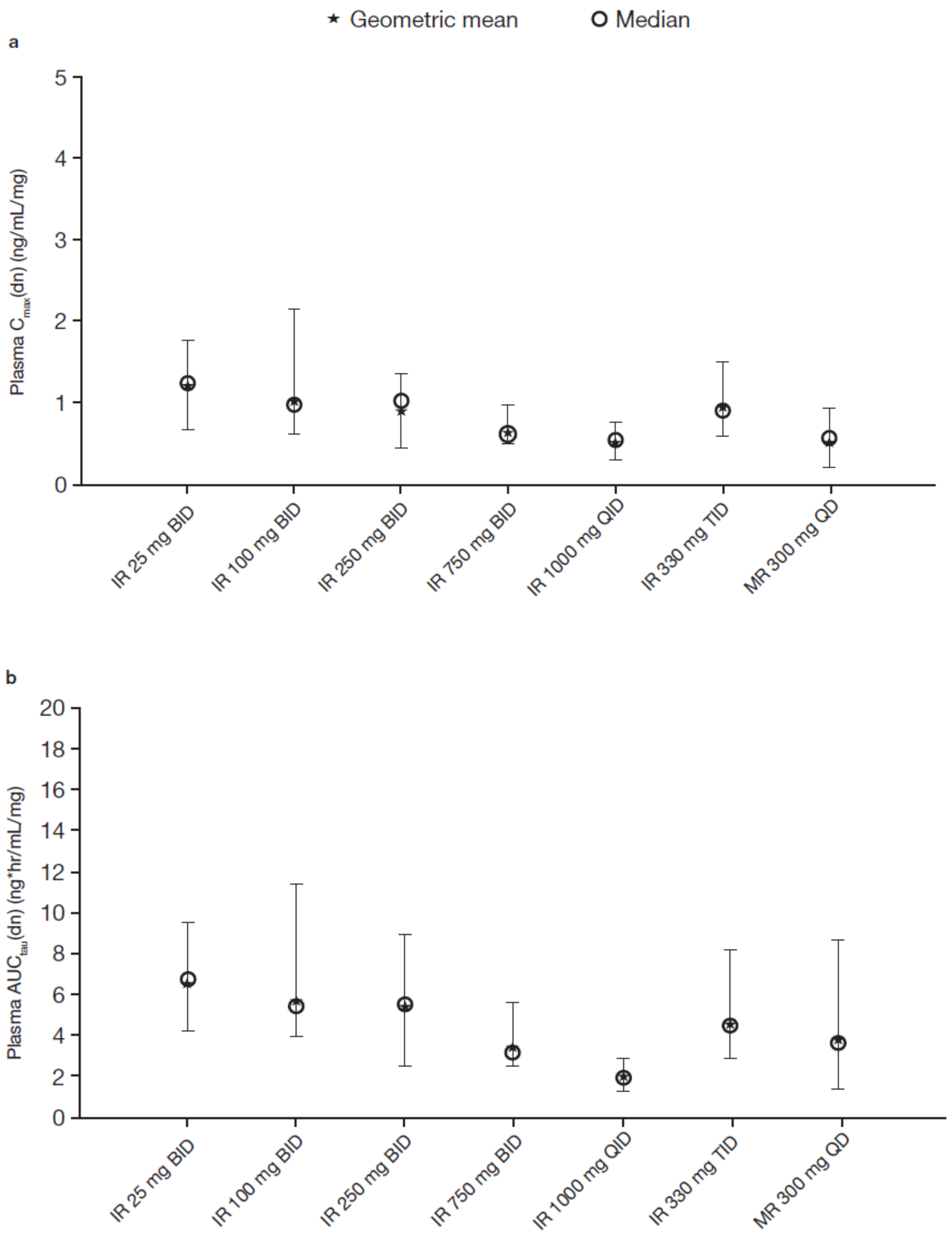

Dose normalized to a 1 mg dose

The geometric mean is calculated for each dose group; stars represent geometric means

Bars represent the range of minimum and maximum values

All doses were administered orally under fed conditions (standard meal)

AUC area under the concentration-time profile curve, AUC_{tau} AUC from time 0 to time tau, the dosing interval, where tau = 6, 8, 12, and 24 hours for QID, TID, BID, and QD dosing, respectively, *BID* twice daily, C_{max} maximum observed concentration, *dn* dose-normalized, *IR* immediate-release, *MAD* multiple ascending doses, *MR* modified-release, *QD* once daily, *QID* four times per day, *TID* three times per day
